# Expression of p11 in Patients with Depression

**DOI:** 10.3390/jcm11195743

**Published:** 2022-09-28

**Authors:** Małgorzata Gałecka, Katarzyna Bliźniewska-Kowalska, Piotr Gałecki, Janusz Szemraj, Agata Orzechowska

**Affiliations:** 1 Department of Psychotherapy, Medical University of Lodz, 91-229 Lodz, Poland; 2 Department of Adult Psychiatry, Medical University of Lodz, 91-229 Lodz, Poland; 3 Department of Medical Biochemistry, Medical University of Lodz, 92-215 Lodz, Poland

**Keywords:** depression, p11, gene expression

## Abstract

(1) Background: Some studies suggest that the p11 protein, belonging to the so-called S100 family and located, i.a., in the nucleus accumbens of the brain, is responsible for the occurrence of depression. This protein is encoded by the S100A10 gene. The aim of our study was to evaluate the expression of the S100A10 gene at the mRNA and protein levels in patients with depressive disorders and to determine the impact of p11 in the etiopathogenesis of depression; (2) Methods: A total of 290 people (190 depressed patients, 100 healthy controls) participated in the study. Socio-demographic and clinical data were collected. The severity of depressive symptoms was assessed using the Hamilton Depression Rating Scale (HDRS). Venous blood was collected from all participants. RT-PCR was used to evaluate gene expression at the mRNA level, while enzyme-linked immunosorbent assay (ELISA) was used to evaluate gene expression at the protein level; (3) Results: The results indicate slightly increased S100A10 gene expression (both at the protein and mRNA levels) in patients with depression, but these values do not reach statistical significance; (4) Conclusions: Due to the fact that the study was limited by the participation of patients already undergoing antidepressant treatment, its results may confirm that pharmacological treatment affecting serotonin neurotransmission is effective in upregulation of p11 in patients with depression.

## 1. Introduction

The number of people diagnosed with depression worldwide is increasing every year. The lifetime risk of developing severe depression is around 20–25% in women and 12–17% in men. Despite their high prevalence, the etiopathogenesis of depressive disorders has not yet been fully understood. Due to the fact that solely around two-thirds of patients suffering from depression respond to the standard antidepressant treatment, it is crucial to search for potential pathomechanistic and contributory factors to treatment resistance [1,2,3].

Current data indicate that depression is a disorder of multifactorial aetiology. Researchers pay attention to theories related to the lack of proper regulation of neurotransmission in the brain and the impact of the immune system on the development of depressive disorders. Traumatic life experiences, especially those characterized by chronic stress, are factors causing a cascade of biological changes, mainly pro-inflammatory in their nature [4,5].

Nowadays, owing to the development of science and technological capabilities, more and more research focuses on the genetic background of diseases, including mental disorders. So far, no single gene has been discovered to account for the mechanism of depression. Many studies emphasize the importance of epigenetic changes in the development of depressive disorders, which in turn also points out the significance of environmental factors in triggering a potential predisposition to developing depression [6].

In this search for potential genetic predisposition to depressive disorders, the p11 protein caught the attention of scientists. It is encoded by the S100A10 gene. Many hypothesize that this gene, belonging to the so-called S100 gene family and located, i.a., in the nucleus accumbens of the brain, is responsible for the occurrence of depression [7,8]. The aim of our study was to evaluate the expression of the S100A10 gene at the mRNA and protein levels in patients with depressive disorders and to determine the impact of p11 in the etiopathogenesis of depression.

## 2. Materials and Methods

### 2.1. Characteristics of Study Participants

A total of 290 participants took part in the study, of which190 were patients with depressive disorders (diagnosis F32 and F33 according to ICD-10) [9] and the control group consisted of 100 healthy volunteers. The groups did not differ significantly statistically in terms of gender (*p* = 0.4583). Women’s predominance was present in both groups—117 and 66 females in the study and control groups, respectively. Patients from the study group were older (47.51 ± 11.18 years) than the volunteers from the control group (29.36 ± 8.71 years) (*p* < 0.001). However, no relationship was found between age and expression in the test group and the control group (presented in Results section).

The mental status of all participants was assessed by a qualified psychiatrist on the day of admission to the study. The exclusion criteria were as follows: psychiatric diagnosis other than depressive disorders, major neurological or somatic diseases (including autoimmune diseases and presence of acute infection), and substance abuse or addiction. All included participants were of Polish origin. Patients with depressive disorders were undergoing pharmacological antidepressant treatment. Participation in the study was voluntary and was not related to any change in pharmacological treatment. Written informed consent was obtained from each patient in accordance with the study protocol approved by the Bioethics Committee of the Medical University of Lodz (No. RNN/833/11/KB).

Demographics were obtained from all study participants. Data regarding the course of depressive disorder were collected using the Composite International Diagnostic Interview (CIDI) [10], also taking into account the duration of the disease (in years), the number of depressive episodes, and the number of psychiatric hospitalizations. The severity of depressive symptoms was assessed using the Polish adaptation of the 17-point Hamilton Depression Rating Scale (HDRS) [11]. Cronbach’s alpha (reliability of the tau equivalent) for this scale was 0.70; the sensitivity coefficient was 0.78, and the test validity coefficient was 0.75 [11,12].

Table 1 presents the clinical characteristics of study group.

### 2.2. Molecular Analysis

#### 2.2.1. Determination of Protein Concentration

Testing of the samples, as well as the reference samples, was always performed in parallel in triplicate. Reaction mixture was added to wells containing serum. The absorbance of the samples was measured using a Multiskan Ascent microplate photometer (Thermo Labsystems, Waltham, MA, USA) and the total protein concentration was calculated from the standard curve equation.

#### 2.2.2. Enzyme-Linked Immunosorbent Assay (ELISA)

The concentration of p11 protein in patient serum was determined using the Human S 100 calcium binding protein A10 ELISA Kit (MyBioSource, San Diego, CA, USA) according to the protocols provided by the manufacturer. β-actin was used as an endogenous control for protein concentration in the samples and assayed using the Human Actin Beta (ACTb) ELISA Kit (BMASSAY) based on the manufacturer’s recommendations. Analytical curves were performed for the analysed proteins to determine protein concentration.

#### 2.2.3. Isolation of Total RNA

Isolation of total RNA from patients’ blood was performed using the InviTrap Spin Universal RNA Kit (Stratec molecular, Berlin, Germany) based on the manufacturer’s recommendations. Absorbance was measured using a spectrophotometer (Picodrop).

#### 2.2.4. Quality Analysis of Isolated RNA

The quality of total RNA was checked using the Agilent RNA 6000 Nano Kit (Agilent Technologies) according to the manufacturer’s recommendations. The quality of the isolated RNA was checked using a 2100 Bioanalyser (Agilent Technologies, Santa Clara, CA, USA). The level of degradation of total RNA was determined by electrophoregram and RIN values were recorded. Only samples with RIN values > 7 were analysed further.

#### 2.2.5. Reverse Transcription RT-PCR

The RT reaction was performed using the TaqMan^®^ RNA Reverse Transcription Kit (Applied Biosystems) based on the manufacturer’s recommendations, using the specific probes Hs00751478_s1 and Hs04194366_g1 for the p11 and RPL13A genes, respectively, provided by Applied Biosystems.

#### 2.2.6. Real-Time PCR Reaction—Scanning the miRNA Panel

The real-time PCR reaction was performed using TaqMan^®^ Universal PCR Master Mix, No UNG (Applied Biosystems, Waltham, MA, USA) according to the protocol provided by the manufacturer. The comparative Ct method [13] was used to calculate the relative expression of miRNA genes. The expression level of the p11 gene in each tissue was normalised against the reference gene RPL13A. Fluorescence emission data were captured and mRNA levels were quantified using critical threshold (Ct) values. Analyses were performed using ABI Prism 7000 software. Controls without RT and without cDNA template were performed at each assay. Relative gene expression levels were obtained using the ∆∆Ct calculation of standard 2-∆∆ct and expressed as fold change from the control sample [13]. Amplification-specific transcripts were further confirmed by obtaining melting curve profiles.

### 2.3. Statistical Analysis

All statistical procedures were set as two-tailed. Generalized linear models (GLM) with robust standard errors were performed to test differences in numerical traits between the studied groups. All the models were controlled for the participants’ age and gender. For nonparametric measures a chi-squared test was used. There were computed correlation coefficients: for variables measured on the same scale (Pearson correlation coefficient) and for traits measured on various scales (Spearman’s rank correlation coefficient). A level of *p* < 0.05 was found to be statistically significant.

## 3. Results

**Figure 1:**
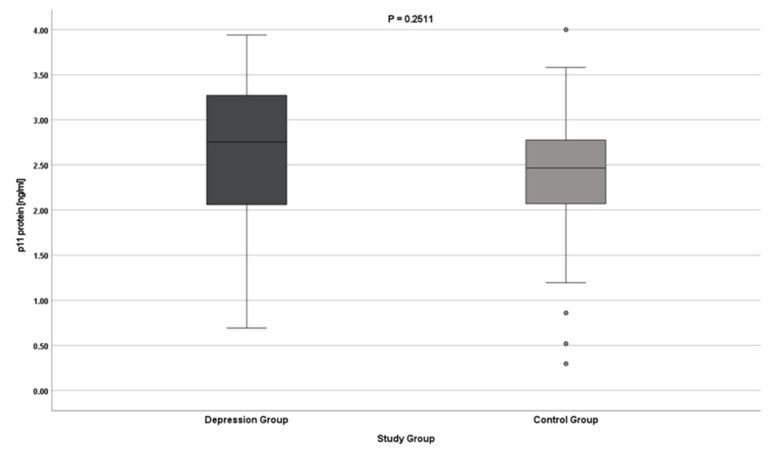
p11 gene expression at the protein level.

**Figure 2:**
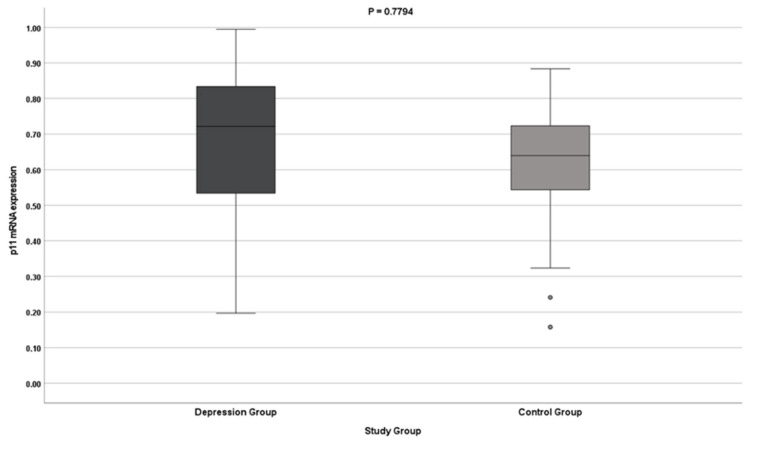
p11 gene mRNA expression.

The results indicate no differences in the expression of the S100A10 gene (both at the protein and mRNA levels) in patients with depression. The results do not reach statistical significance (Table 2, Figure 1 and Figure 2).

In the group of depressed patients, the correlation between p11 gene expression and clinical variables was calculated, including the severity of depression symptoms measured with the HDRS scale, number of hospitalizations, duration of the disease (in years), and the number of depressive episodes (Table 3).

Although the studied patients were significantly older than the controls, no statistically significant correlation was observed between age and the expression of the p11 gene in the test and control groups (Table 4).

## 4. Discussion

The p11 protein, also known as S100 calcium-binding protein A10 (S100A10), is a member of the S100 family of proteins containing two EF-hand calcium-binding motifs. S100 proteins are present in the cytoplasm and nucleus of many cell types. They are responsible for the regulation of cell cycle progression and differentiation [14,15]. The p11 protein is also said to play an essential role in the serotonin signalling regulation in the CNS.

This protein interacts with the serotonin (5-HT) receptors and modulates the receptor signal transduction pathways activated by the binding of serotonin. It also recruits the cell surface expression of the 5-HT4 receptor, increasing its concentration at the synapse, which in turn results in increased serotonin neurotransmission [16,17,18]. In studies performed on animal models, p11 and 5-HTR4 are coexpressed in brain regions associated with depression [17,18,19,20]. Research also indicates the interaction of p11 with a wide range of serotonin receptors, such as 5-HT 1B (5-HT1BR), 5-HT1DR, and as previously mentioned, 5-HT4R [21,22].

Immunohistochemical studies and in situ hybridization in mouse and human brains revealed that p11 is expressed in GABAergic and cholinergic interneurons and in monoaminergic, cholinergic, glutaminergic, and GABAergic projection neurons [23,24]. However, research shows that p11 is not only found in neurons but is also in other tissues like in epithelial and endothelial cells [25,26].

Post-mortem studies show a decreased expression of p11 protein in the brain tissues of depressed individuals [22,27]. At the same time, Svenningsson et al. noted that p11 expression increases in neural tissues of rodents undergoing antidepressant treatment [22]. This may support the crucial role of p11 as a mediator of antidepressant response. This seems to be particularly important in relation to such a large group of patients suffering from TRD (treatment-resistant depression). The main limitation of the currently available antidepressant therapies is an often quite long waiting period for a symptomatic effect in depressed patients. Activation of 5-HTR4 causes a rapid antidepressant response in models of depression in rodents [28]. However, this mechanism still requires careful research to be introduced as a new treatment route.

The results of our research suggest an unchanged level of p11 expression in depressive patients, which is not in line with the general hypothesis assuming a decreased level of p11 in depressive patients. However, Rothermundt et al. stated that the level of S-100B, another S100 family member, is increased in melancholic but not in non-melancholic major depression [29]. The limitation of our study is also that patients were already undergoing the antidepressant treatment, which may have a great impact on p11 expression.

Inflammation is recognized as one of the most important contributory factors to depression. Although cytokines appear to be potent regulators of p11, it is not yet clear whether they directly increase p11 production, or whether they act indirectly by influencing the production and signalling of neurotrophic factors such as BDNF. In addition, one study indicates that exposure to the synthetic glucocorticoid dexamethasone increases p11 mRNA expression in the prefrontal cortex by activating glucocorticoid response elements in the p11 promoter. The increase in p11 levels observed in a mouse model in response to stress or exposure to glucocorticoids may be a physiological response to counteracting depression symptoms by increasing p11 levels and thus serotonin sensitivity. Despite the available studies, the above data do not confirm that p11 is regulated by BDNF, cytokines, and glucocorticoids, critical factors in the pathogenesis of mood disorders [30].

## 5. Conclusions

The results are partially consistent with previously published studies. Due to the fact that the study was limited by the participation of patients already undergoing antidepressant treatment, further studies need to confirm whether pharmacological treatment affecting serotonin neurotransmission is effective in the upregulation of p11 in patients with depression.

## Figures and Tables

**Table 1:** Clinical characteristics of study group.

Analysed Trait	Statistical Parameters *			
	M	Me	SD	Min.–Max.
Number of hospitalizations ^1^	2.01	1	2.00	0–12
Disease duration time (years)	6.18	4	7.05	1–40
Number of episodes	4.56	2	5.33	1–20
HDRS *	22.82	23	6.86	1–51

* Explanations of abbreviations used in the table: M—mean; Me—median; SD—standard deviation, HDRS—Hamilton Depression Rating Scale; ^1^ Number of hospitalizations before the current hospitalization (onset of the study).

**Table 2:** Detailed descriptive statistics for p11 gene expression by study group.

Gene	Study Group	Statistical Parameter							
		M	Me	Q_1_–Q_3_ (IQR)	SD	SE	95% CI	Min.–Max.	*p*
p11 protein [ng/mL]	Test group	2.583	2.755	2.057–3.270 (1.213)	0.781	0.057	2.471–2.695	0.690–3.940	0.2511
	Control group	2.384	2.464	2.069–2.780 (0.711)	0.614	0.061	2.262–2.506	0.298–3.999	
	Overall	2.514	2.560	2.060–3.160 (1.100)	0.733	0.043	2.430–2.599	0.298–3.999	
p11 (S100A10) mRNA [2^−∆CT^]	Test group	0.669	0.722	0.534–0.835 (0.301)	0.190	0.014	0.642–0.696	0.197–0.995	0.7794
	Control group	0.625	0.639	0.544–0.724 (0.180)	0.140	0.014	0.597–0.652	0.158–0.884	
	Overall	0.654	0.662	0.537–0.816 (0.279)	0.175	0.010	0.633–0.674	0.158–0.995	

Explanations of abbreviations used in result tables: M—mean; Me—median; Q—quartile; Q1—first quartile; Q3—third quartile; IQR—interquartile range; SD—standard deviation; SE—standard error; CI—confidence interval; Min—minimum; Max—maximum; *p*—statistical significance of differences by study groups: All empirical data, considering the gene expression, were log transformed before testing the hypothesis. All models carried out were controlled for age and gender.

**Table 3:** Spearman’s rho correlations of p11 gene expression with clinical variables in the test group (depression).

		p11 Protein	p11 mRNA
Number of hospitalizations	Correlation coefficient	0.012	0.023
	Sig. (2-tailed)	0.8731	0.7508
	N	186	186
Disease duration (years)	Correlation coefficient	0.030	0.046
	Sig. (2-tailed)	0.6795	0.5319
	N	186	186
Number of episodes	Correlation coefficient	0.003	0.013
	Sig. (2-tailed)	0.9641	0.8557
	N	186	186
HDRS	Correlation coefficient	0.058	0.051
	Sig. (2-tailed)	0.4369	0.4884
	N	184	184

**Table 4:** Correlation of p11 gene expression with age (by study group).

		p11 Protein	p11 mRNA
Overall	Correlation coefficient	0.124 *	0.135 *
	Sig. (2-tailed)	**0.0350**	**0.0220**
	N	**289**	**289**
Test group (depression)	Correlation coefficient	0.080	0.092
	Sig. (2-tailed)	0.2729	0.2054
	N	190	190
Control group	Correlation coefficient	−0.162	−0.138
	Sig. (2-tailed)	0.1085	0.1737
	N	99	99

* statistically significant differences are marked in bold.

## Data Availability

The data that support the findings of this study are available upon request from author M.G.
